# Supplementary material for: Analysis of Teachers’ Visual Behaviour in Classes: A Systematic Review
Source: Eur J Investig Health Psychol Educ. 2025 Apr 5;15(4):54. doi: 10.3390/ejihpe15040054 (PMC12025623; doi:10.3390/ejihpe15040054)
Supplement: Supplementary file 1 [file ejihpe-15-00054-s001.zip › ejihpe-3471374-supplementary.pdf]

Supplementary Materials

Table S1: Reason for excluding the articles identified in the first "Screening" phase.

Year	Author(s)	Title	Reason for Exclusion
2025	Huang et al.	Enhancing preservice teachers' noticing via adaptive feedback in a virtual reality classroom	Published in 2025
2024	ben Chikha et al.	Eye-Tracking Analyses of a Coach's Pointing Gestures Timed With Speech: Implications for Players' Recall of Basketball Tactical Instructions	Eye tracking apparatus for other purpose
2024	ben Chikha et al.	The role of coach's gaze guidance on memorization of tactical movements in basketball: an eye tracking study	Eye tracking apparatus for other purpose
2024	Brückner & Zlatkin-Troitschanskaia	Effects of teacher students' study progress on their gaze behavior while solving of an economics knowledge test	Eye tracking apparatus for other purpose
2024	Burch & Kurzhals	Teaching Eye Tracking: Challenges and Perspectives	Descriptive study
2024	Davies et al.	Acceptability and Feasibility of Portable Eye-Tracking Technology within a Children's Dynamic Sport Context: An Exploratory Study with Boys Who Play Grassroots Football	Eye tracking apparatus for other purpose
2024	Donmez & Cagiltay	Eye training games for children with low vision: A design-based research study	Eye tracking apparatus for other purpose
2024	Eaton et al.	Impaired Social Attention and Cognitive Empathy in a Paediatric Sample of Children with Symptoms of Anxiety	Eye tracking apparatus for other purpose
2024	Griffiths et al.	The ability of typically developing 2–3 year olds to infer the control mechanism for eye-gaze technology and the impact of causal language instruction	Students' visual behaviour as the dependent variable
2024	Haataja et al.	Student visual attention during group instruction phases in collaborative geometry problem solving	Students' visual behaviour as the dependent variable
2024	Hsieh et al.	Feasibility of an eye-gaze technology intervention for students with severe motor and communication difficulties in Taiwan	Eye tracking apparatus for other purpose
2024	Huangfu et al.	An Eye-Tracking Study on the Effects of Displayed Teacher Enthusiasm on Students' Learning Procedural Knowledge of Chemistry in Video Lectures	Students' visual behaviour as the dependent variable
2024	Junior et al.	Use of eye tracking in medical education	Descriptive study
2024	Kartal & Okyar	A bibliometric analysis of eye-tracking technology in second language research	Descriptive study
2024	Keskin et al.	Eye-tracking research on teacher professional vision: A meta-analytic review	Descriptive study
2024	Khalid et al.	InerSens: A Block-Based Programming Platform for Learning Sensor Data Analytics in Construction Engineering Programs	Does not use an eye tracking apparatus
2024	Kuang et al.	Instructor's Direct Gaze Not Body Orientation Affects Learning	Students' visual behaviour as the dependent variable
2024	Langner & Graulich	From sight to insight—reflection processes in an eye-gaze-augmented retrospective	Students' visual behaviour as the dependent variable
2024	Li	Eye-Tracking Research in Interactive Language Learning Environments: A Systematic Review	Descriptive study
2024	Liang et al.	Teaching expectancy improves video-based learning: Evidence from eye-movement synchronization	Students' visual behaviour as the dependent variable
2024	Lim et al.	Leveraging machine learning techniques for student's attention detection: a review	Descriptive study
2024	Meister et al.	Top-down and bottom-up effects on visual perception during data-based scientific reasoning in the context of population dynamics	Eye tracking apparatus for other purpose
2024	Michalik	Enhancing Archaeological Teaching Through Eye-Tracking: A Pilot Study on Eye Movement Modelling Examples and Teaching Artefact Analysis	Descriptive study
2024	Muthu et al.	Visual interpretation of clinical images among dental students using eye-tracking technology	Students' visual behaviour as the dependent variable

2024	Panjaburee et al.	Effects of a personalized game on students' outcomes and visual attention during digital citizenship learning	Students' visual behaviour as the dependent variable
2024	Pham et al.	Investigation of the influences of instructors and different media on learning attention with a wearable eye-tracking system in the physical classrooms	Students' visual behaviour as the dependent variable
2024	Rau et al.	Understanding Collaborative Learning of Molecular Structures in AR with Eye Tracking	Students' visual behaviour as the dependent variable
2024	Shi et al.	The impacts of instructor's visual attention and lecture type on students' learning performance and perceptions	Students' visual behaviour as the dependent variable
2024	Smirnova	Eye-tracking is a study of the features of the perceptual activity of preschoolchildren with hearing impairment wheninteracting with visual educationalmaterial in the learning process	Students' visual behaviour as the dependent variable
2024	Wang	Mind Wandering in Videos That Integrate Instructor's Visuals: An Eye Tracking Study	Students' visual behaviour as the dependent variable
2024	Witt et al.	Eye-tracking research on teachers' professional vision: A scoping review	Descriptive study
2024	Yu et al.	Autistic preschoolers display reduced attention orientation for competition but intact facilitation from a parallel competitor: Eye-tracking and behavioral data	Students' visual behaviour as the dependent variable
2024	Zhang et al.	How does drawing influence the effectiveness of oral self-explanation versus instructional explanation in video learning?	Students' visual behaviour as the dependent variable
2023	Alkabbany et al.	An Experimental Platform for Real-Time Students Engagement Measurements from Video in STEM Classrooms	Students' visual behaviour as the dependent variable
2023	Behnke	Which Factors Influence Learners' Visual Attention to Images in Geographic Learning Media?	Students' visual behaviour as the dependent variable
2023	Härkki	Mobile gaze tracking and an extended linkography for collaborative sketching and designing	Students' visual behaviour as the dependent variable
2023	Lee et al.	Engagement and effectiveness of symbolic and iconic learning support for math problem representation: an eye tracking study	Eye tracking apparatus for other purpose
2023	Lian et al.	An eye-tracking study on autistic children's visual attention: The use of spatial-progression, time-sequence, colours and shape-patterns in picture book designs	Eye tracking apparatus for other purpose
2023	Lian et al.	The effect of background elements of pictures on the visual attention among ASD children with intellectual disabilities, children with intellectual disabilities and typical development: Evidence from eye-tracking and fMRI	Eye tracking apparatus for other purpose
2023	Liberman & Dubovi	The Effect of the Modality Principle to Support Learning with Virtual Reality: An Eye-Tracking and Electrodermal Activity Study	Students' visual behaviour as the dependent variable
2023	Olla et al.	The Differential Effects of Feedback Provision on Students' Performance on Test-Based Assessments	Students' visual behaviour as the dependent variable
2023	Puimège et al.	Promoting L2 Acquisition of Multiword Units through Textually Enhanced Audiovisual Input: An Eye-Tracking Study	Students' visual behaviour as the dependent variable
2023	Schweinberger et al.	Eye tracking as feedback tool in physics teacher education	Students' visual behaviour as the dependent variable
2023	Shadiev & Li	A review study on eye-tracking technology usage in immersive virtual reality learning environments	Descriptive study
2023	Shang et al.	The effect of structured stepwise presentations on students' fraction learning: an eye-tracking study	Students' visual behaviour as the dependent variable
2023	Smirnova	Eye tracking study of visual attention of children with hearing impairments in a learning situation	Students' visual behaviour as the dependent variable
2023	Su et al.	Own-Age Effects in a Face-Emotion Recognition Intervention for Children With ASD--Evidence From Eye Movements	Eye tracking apparatus for other purpose
2023	Susac et al.	Linking information from multiple representations: an eye-tracking study	Students' visual behaviour as the dependent variable
2023	Teo & Peh	An exploratory study on eye-gaze patterns of experts and novices of science inference graph items	Eye tracking apparatus for other purpose

2023	Wong et al.	Investigating the Relationship between Visual Attention, Story Comprehension, and Vocabulary Skills in Malaysian Prereaders: An Eye-Tracking Study	Students’ visual behaviour as the dependent variable
2023	Xia et al.	The effects of information format on learners’ experience	Students’ visual behaviour as the dependent variable
2023	Yang & Wang	Tracking visual attention during learning of complex science concepts with augmented 3D visualizations	Students’ visual behaviour as the dependent variable
2023	Zohar et al.	Predictive Eye Movements Characterize Active, Not Passive, Participation in the Collective Embodied Learning of a Scientific Concept	Students’ visual behaviour as the dependent variable
2022	Asish et al.	Detecting distracted students in educational VR environments using machine learning on eye gaze data	Students’ visual behaviour as the dependent variable
2022	Chiou et al.	Exploring How Students Interact with Guidance in a Physics Simulation: Evidence from Eye-Movement and Log Data Analyses	Students’ visual behaviour as the dependent variable
2022	Gavric et al.	Tracking Students? Visual Attention During E-Learning in the Time of COVID-19	Students’ visual behaviour as the dependent variable
2022	Hekele et al.	Remote vocational learning opportunities-A comparative eye-tracking investigation of educational 2D videos versus 360° videos for car mechanics	Students’ visual behaviour as the dependent variable
2022	Ji et al.	The role of eye tracker in teaching video-assisted thoracoscopic surgery: the differences in visual strategies between novice and expert surgeons in thoracoscopic surgery	Eye tracking apparatus for other purpose
2022	Jung et al.	The impact of textual enhancement and frequency manipulation on incidental learning of collocations from reading	Students’ visual behaviour as the dependent variable
2022	Li et al.	Detecting preservice teachers’ visual attention under prediction and nonprediction conditions with eye-tracking technology	Eye tracking apparatus for other purpose
2022	Lian et al.	The influence of picture book design on visual attention of children with autism: a pilot study	Eye tracking apparatus for other purpose
2022	Liu et al.	How to reflect more effectively in online video learning: Balancing processes and outcomes	Students’ visual behaviour as the dependent variable
2022	Lui et al.	User Experiences of Eye Gaze Classroom Technology for Children With Complex Communication Needs	Students’ visual behaviour as the dependent variable
2022	Negi & Mitraet al.	Native language subtitling of educational videos: A multimodal analysis with eye tracking, EEG and self-reports	Students’ visual behaviour as the dependent variable
2022	Pi et al.	Modulation of instructor’s eye gaze by facial expression in video lectures	Students’ visual behaviour as the dependent variable
2022	Qian et al.	How teacher enthusiasm affects students’ learning of chemistry declarative knowledge in video lectures	Students’ visual behaviour as the dependent variable
2022	Samudra et al.	Can Small Changes Matter? Reducing Cognitive Load in Educational Media Supports Low-Income Preschoolers’ Vocabulary Learning	Students’ visual behaviour as the dependent variable
2022	Santapuram et al.	Mechanisms by which Early Eye Gaze to the Mouth During Multisensory Speech Influences Expressive Communication Development in Infant Siblings of Children with and without Autism	Eye tracking apparatus for other purpose
2022	Sue et al.	To What Extent Do Pictures Support Malaysian Children’s Comprehension of Stories?	Students’ visual behaviour as the dependent variable
2022	Tsai et al.	What do critical reading strategies look like? Eye-tracking and lag sequential analysis reveal attention to data and reasoning when reading conflicting information	Students’ visual behaviour as the dependent variable
2022	van den Bosch et al.	Teachers’ visual inspection of Curriculum-Based Measurement progress graphs: An exploratory, descriptive eye-tracking study	Eye tracking apparatus for other purpose
2022	van Marlen et al.	Looking through Sherlock’s eyes: Effects of eye movement modelling examples with and without verbal explanations on deductive reasoning	Students’ visual behaviour as the dependent variable
2022	Wang et al.	Examining the effects of students’ self-efficacy and prior knowledge on learning and visual behavior in a physics game	Students’ visual behaviour as the dependent variable
2022	Winke et al.	“So I Think [Arabic character omitted] Is Bear!” An Initial Data-Driven Explanation of How Arabic Students Use Captioned Video to Learn Vocabulary	Students’ visual behaviour as the dependent variable

2021	Ahsan & Obaidellah	Visual behavior on problem comprehension among novice programmers with prior knowledge	Eye tracking apparatus for other purpose
2021	Averbukh	Evolution of human computer interaction	Descriptive study
2021	Banire et al.	Impact of mainstream classroom setting on attention of children with autism spectrum disorder: an eye-tracking study	Eye tracking apparatus for other purpose
2021	Bhagat et al.	Tracking the Process and Motivation of Math Learning with Augmented Reality	Eye tracking apparatus for other purpose
2021	Dovigo et al.	Social and Cognitive Interactions Through an Interactive School Service for RTT Patients at the COVID-19 Time	Students' visual behaviour as the dependent variable
2021	Fujii et al.	Influence of dental education on eye gaze distribution when observing facial profiles with varying degrees of lip protrusion	Eye tracking apparatus for other purpose
2021	Gangopadhyay & Kaushanskaya	Word learning in monolingual and bilingual children: The influence of speaker eye-gaze	Students' visual behaviour as the dependent variable
2021	Grill & Younie	Evaluating Eye Tracking Technology for Assessment of Students with Profound and Multiple Learning Difficulties	Students' visual behaviour as the dependent variable
2021	Heldal et al.	Supporting school aged children to train their vision by using serious games	Students' visual behaviour as the dependent variable
2021	Hochhauser et al.	Investigating attention in young adults with autism spectrum disorder (ASD) using change blindness and eye tracking	Eye tracking apparatus for other purpose
2021	Ishizaki et al.	Eye gaze differences in school scenes between preschool children and adolescents with high-functioning autism spectrum disorder and those with typical development	Eye tracking apparatus for other purpose
2021	Jarodzka et al.	Eye-Tracking in Educational Practice: Investigating Visual Perception Underlying Teaching and Learning in the Classroom	Descriptive study
2021	Madsen et al.	Synchronized eye movements predict test scores in online video education	Students' visual behaviour as the dependent variable
2021	McConnell & Troop-Gordon	Attentional Biases to Bullies and Bystanders and Youth's Coping With Peer Victimization	Eye tracking apparatus for other purpose
2021	Nückles	Investigating Visual Perception in Teaching and Learning with Advanced Eye-Tracking Methodologies: Rewards and Challenges of an Innovative Research Paradigm	Descriptive study
2021	Pouta et al.	Student Teachers' and Experienced Teachers' Professional Vision of Students' Understanding of the Rational Number Concept	Eye tracking apparatus for other purpose
2021	Puurtinen et al.	Investigating visual attention toward foods in a salad buffet with mobile eye tracking	Eye tracking apparatus for other purpose
2021	Rusch	Combining fMRI and Eye-tracking for the Study of Social Cognition	Descriptive study
2021	Tsai & Wu	Visual search patterns, information selection strategies, and information anxiety for online information problem solving	Students' visual behaviour as the dependent variable
2021	Wang et al.	Towards Collaborative and Intelligent Learning Environments Based on Eye Tracking Data and Learning Analytics: A Survey	Descriptive study
2021	Woolverton & Pollastri	An Exploration and Critical Examination of How "Intelligent Classroom Technologies" Can Improve Specific Uses of Direct Student Behavior Observation Methods	Students' visual behaviour as the dependent variable
2021	Wu et al.	The Development of Visual Expertise in ECG Interpretation: An Eye-Tracking Augmented Re Situ Interview Approach	Students' visual behaviour as the dependent variable
2021	Yang et al.	Students' achievement motivation moderates the effects of interpolated pre-questions on attention and learning from video lectures	Students' visual behaviour as the dependent variable
2021	Zimmermann et al.	Seeing things differently: Gaze shapes neural signal during mentalizing according to emotional awareness	Eye tracking apparatus for other purpose
2020	Childers et al.	Does children's visual attention to specific objects affect their verb learning?	Students' visual behaviour as the dependent variable
2020	Emerson et al.	Multimodal learning analytics for game-based learning	Students' visual behaviour as the dependent variable
2020	Feng et al.	Virtual pointer for gaze guidance in laparoscopic surgery	Eye tracking apparatus for other purpose
2020	Guarino & Wakefield	Teaching Analogical Reasoning With Co-speech Gesture Shows Children Where to Look, but Only Boosts Learning for Some	Students' visual behaviour as the dependent variable

2020	Harvanová et al.	Pohyby očí, selektívna pozornosť a profesijné videnie učiteľov a učiteliek	Published in Czech
2020	Iannizzotto et al.	Remote Eye-Tracking for Cognitive Telerehabilitation and Interactive School Tasks in Times of COVID-19	Students' visual behaviour as the dependent variable
2020	Klein et al.	Visual attention while solving the test of understanding graphs in kinematics: an eye-tracking analysis	Students' visual behaviour as the dependent variable
2020	Liao et al.	Electronic storybook design, kindergartners' visual attention, and print awareness: An eye-tracking investigation	Students' visual behaviour as the dependent variable
2020	McEwen et al.	Interlocutors and Interactions: Examining the Interactions Between Students With Complex Communication Needs, Teachers, and Eye-Gaze Technology	Students' visual behaviour as the dependent variable
2020	Nehring & Busch	Setting up Chemistry Demonstrations According to the Left-to-Right Principle: An Eye-Movement-Pattern-Based Analysis	Descriptive study
2020	Perfect et al.	A systematic review investigating outcome measures and uptake barriers when children and youth with complex disabilities use eye gaze assistive technology	Descriptive study
2020	Pi et al.	Instructor presence in video lectures: Eye gaze matters, but not body orientation	Students' visual behaviour as the dependent variable
2020	Salmerón et al.	Using eye-movement modelling examples to improve critical reading of multiple webpages on a conflicting topic	Students' visual behaviour as the dependent variable
2020	Samudra et al.	Is attention the missing link? Coviewing and preschoolers' comprehension of educational media	Students' visual behaviour as the dependent variable
2020	Sharma et al.	Webcam controlled robotic arm for persons with SSMI	Eye tracking apparatus for other purpose
2020	Shirley et al.	Impact of an educational intervention on eye gaze behaviour in retinal image interpretation by consultant and trainee ophthalmologists	Eye tracking apparatus for other purpose
2020	Valle et al.	Effects of Anticipation Guide Use on Visual Attention Distribution in a Multimedia Environment: An Eye Tracking Study	Eye tracking apparatus for other purpose
2020	Wang et al.	Does visual attention to the instructor in online video affect learning and learner perceptions? An eye-tracking analysis	Students' visual behaviour as the dependent variable
2020	Zimmermann et al.	Visual Behaviour Strategies of Operators during Catheter-Based Cardiovascular Interventions	Eye tracking apparatus for other purpose
2019	Beach et al.	How Do Elementary Teachers Study and Learn from a Multimedia Model of Reading Development? An Exploratory Eye-Tracking Study	Eye tracking apparatus for other purpose
2019	Billeci et al.	Emotional processing deficits in Italian children with Disruptive Behavior Disorder: The role of callous unemotional traits	Eye tracking apparatus for other purpose
2019	del Campos et al.	Effect of body experiences on the perceptual pattern and judgment in ballet: a cases study in classic dance	Eye tracking apparatus for other purpose
2019	Gottschling et al.	Readers' Processing and Use of Source Information as a Function of Its Usefulness to Explain Conflicting Scientific Claims	Eye tracking apparatus for other purpose
2019	He et al.	The characteristics of intelligence profile and eye gaze in facial emotion recognition in mild and moderate preschoolers with autism spectrum disorder	Students' visual behaviour as the dependent variable
2019	Kao et al.	Reading behavior and the effect of embedded selfies in role-playing picture e-books: An eye-tracking investigation	Students' visual behaviour as the dependent variable
2019	Lee & Doherty	Native and Nonnative Processing of Active and Passive Sentences: The Effects of Processing Instruction on the Allocation of Visual Attention	Students' visual behaviour as the dependent variable
2019	Wang et al.	The instructor's gaze guidance in video lectures improves learning	Students' visual behaviour as the dependent variable
2019	Yusof et al.	Graphic novels: Understanding how fifth graders read literartext through eye movement analysis	Students' visual behaviour as the dependent variable
2018	Anderson et al.	Revisiting the Jezebel Stereotype	Descriptive study
2018	Audrin et al.	More Than Meets the Eye: The Impact of Materialism on Information Selection During Luxury Choices	Eye tracking apparatus for other purpose
2018	Molina et al.	Evaluating multimedia learning materials in primary education using eye tracking	Descriptive study

2018	Moreno-Esteva et al.	Application of mathematical and machine learning techniques to analyse eye tracking data enabling better understanding of children's visual cognitive behaviours	Students' visual behaviour as the dependent variable
2018	Scherf et al.	Improving sensitivity to eye gaze cues in autism using serious game technology: study protocol for a phase I randomised controlled trial	Eye tracking apparatus for other purpose
2018	Skibbe et al.	Preschoolers' Visual Attention during Electronic Storybook Reading as Related to Different Types of Textual Supports	Students' visual behaviour as the dependent variable
2018	Stull et al.	An eye-tracking analysis of instructor presence in video lectures	Students' visual behaviour as the dependent variable
2018	Troop-Gordon et al.	Visual Attention to Dynamic Scenes of Ambiguous Provocation and Children's Aggressive Behavior	Eye tracking apparatus for other purpose
2018	Wakefield et al.	Gesture Helps Learners Learn, but Not Merely by Guiding Their Visual Attention	Students' visual behaviour as the dependent variable
2017	Bi & Reid	Evaluating students' understanding of statics concepts using eye gaze data	Students' visual behaviour as the dependent variable
2017	Molina et al.	Applying eye tracking techniques for evaluating learning materials in Primary Education	Eye tracking apparatus for other purpose
2017	Roach et al.	Time limits in testing: An analysis of eye movements and visual attention in spatial problem solving	Eye tracking apparatus for other purpose
2017	van der Gijp et al.	How Visual Search Relates to Visual Diagnostic Performance: A Narrative Systematic Review of Eye-Tracking Research in Radiology	Descriptive study
2017	Yang	Examining the reasoning of conflicting science information from the information processing perspective-an eye movement analysis	Students' visual behaviour as the dependent variable
2016	Browning et al.	The use and limits of eye-tracking in high-fidelity clinical scenarios: A pilot study	Students' visual behaviour as the dependent variable
2016	Manzanares et al.	A Protocol for Recording Visual and Motor Behaviour for Scientific Support in the Teaching and Training of Sailing in a Simulator	Descriptive study
2016	Nussenbaum & Amso	An Attentional Goldilocks Effect: An Optimal Amount of Social Interactivity Promotes Word Learning from Video	Students' visual behaviour as the dependent variable
2016	Pi & Hong	Learning process and learning outcomes of video podcasts including the instructor and PPT slides: a Chinese case	Students' visual behaviour as the dependent variable
2015	Elmadani et al.	Investigating student interactions with tutorial dialogues in EER-Tutor	Students' visual behaviour as the dependent variable
2015	Hua et al.	On semantic-instructed attention: From video eye-tracking dataset to memory-guided probabilistic saliency model	Eye tracking apparatus for other purpose
2015	Najar et al.	Eye tracking and studying examples: how novices and advanced learners study SQL examples	Students' visual behaviour as the dependent variable
2015	Scheiter & Eitel	Signals foster multimedia learning by supporting integration of highlighted text and diagram elements	Students' visual behaviour as the dependent variable
